# Uptake of community‐ versus clinic‐based antiretroviral therapy dispensing in the Central Chronic Medication Dispensing and Distribution program in South Africa

**DOI:** 10.1002/jia2.25877

**Published:** 2022-01-25

**Authors:** Ingrid V. Bassett, Joyce Yan, Sabina Govere, Anele Khumalo, Nompumelelo Ngobese, Zinhle Shazi, Mpilonhle Nzuza, Bridget A. Bunda, Nafisa J. Wara, Ashley Stuckwisch, Dani Zionts, Nduduzo Dube, Sandile Tshabalala, Laura M. Bogart, Robert A. Parker

**Affiliations:** ^1^ Massachusetts General Hospital Division of Infectious Diseases Boston Massachusetts USA; ^2^ Massachusetts General Hospital Medical Practice Evaluation Center Boston Massachusetts USA; ^3^ Center for AIDS Research (CFAR) Harvard University Boston Massachusetts USA; ^4^ Harvard Medical School Boston Massachusetts USA; ^5^ Massachusetts General Hospital Biostatistics Center Boston Massachusetts USA; ^6^ AIDS Healthcare Foundation Durban South Africa; ^7^ South Africa Department of Health Province of KwaZulu‐Natal South Africa; ^8^ RAND Corporation Santa Monica California USA

**Keywords:** HIV care continuum, ARV, community, differentiated care, Africa, cohort studies

## Abstract

**Introduction:**

South Africa's government‐led Central Chronic Medication Dispensing and Distribution (CCMDD) program offers people living with HIV the option to collect antiretroviral therapy at their choice of community‐ or clinic‐based pick‐up points intended to increase convenience and decongest clinics. To understand CCMDD pick‐up point use among people living with HIV, we evaluated factors associated with uptake of a community‐ versus clinic‐based pick‐up point at CCMDD enrolment.

**Methods:**

We collected baseline data from October 2018 to March 2020 on adults (≥18 years) who met CCMDD clinical eligibility criteria (non‐pregnant, on antiretroviral therapy for ≥1 year and virologically suppressed) as part of an observational cohort in seven public clinics in KwaZulu‐Natal. We identified factors associated with community‐based pick‐up point uptake and fit a multivariable logistic regression model, including age, gender, employment status, self‐perceived barriers to care, self‐efficacy, HIV‐related discrimination, and perceived benefits and challenges of CCMDD.

**Results and Discussion:**

Among 1521 participants, 67% were females, with median age 36 years (IQR 30–44). Uptake of a community‐based pick‐up point was associated with younger age (aOR 1.18 per 10‐year decrease, 95% CI 1.05–1.33), being employed ≥40 hours per week (aOR 1.42, 95% CI 1.10–1.83) versus being unemployed, no self‐perceived barriers to care (aOR 1.42, 95% CI 1.09–1.86) and scoring between 36 and 39 (aOR 1.44, 95% CI 1.03–2.01) or 40 (aOR 1.91, 95% CI 1.39–2.63) versus 10–35 on the self‐efficacy scale, where higher scores indicate greater self‐efficacy. Additional factors included more convenient pick‐up point location (aOR 2.32, 95% CI 1.77–3.04) or hours (aOR 5.09, 95% CI 3.71–6.98) as perceived benefits of CCMDD, and lack of in‐clinic follow‐up after a missed collection date as a perceived challenge of CCMDD (aOR 4.37, 95% CI 2.30–8.31).

**Conclusions:**

Uptake of community‐based pick‐up was associated with younger age, full‐time employment, and systemic and structural factors of living with HIV (no self‐perceived barriers to care and high self‐efficacy), as well as perceptions of CCMDD (convenient pick‐up point location and hours, lack of in‐clinic follow‐up). Strategies to facilitate community‐based pick‐up point uptake should be tailored to patients’ age, employment, self‐perceived barriers to care and self‐efficacy to maximize the impact of CCMDD in decongesting clinics.

## 1 INTRODUCTION

South Africa has the largest antiretroviral therapy (ART) program in the world, with 7.6 million people living with HIV (PLWH) and 5.2 million on ART [1]. With universal test and treat, health services in South Africa must adapt to increase coverage while maintaining care for PLWH already on treatment. Public sector clinics face staff shortages, long waiting times and medication stock‐outs [2, 3]. Care retention is suboptimal, partly due to patient dissatisfaction with the overburdened healthcare system and the need to travel to distant clinics [4, 5, 6].

Differentiated service delivery (DSD) models have adapted services to improve outcomes for patients established on ART across South Africa [7, 8]. Adherence clubs, or healthcare worker‐managed groups of PLWH that meet for ART pick‐up, as well as community‐based mobile DSD programs have demonstrated high rates of retention and virologic suppression [9, 10, 11, 12, 13, 14]. Stakeholders have additionally reported cost savings and reduced clinic waiting times with community‐based DSD programs [15, 16]. However, there are limited data from South Africa about patient uptake of fixed medication pick‐up points (PUPs).

The South African Department of Health implemented the Central Chronic Medication Dispensing and Distribution (CCMDD) program in 2014 and has since registered over two million patients for over 30 chronic conditions, with 76% collecting ART [17, 18, 19]. Patients established on ART can be enrolled by their healthcare provider to obtain medication refills at Department of Health‐identified community‐ or clinic‐based PUPs without ongoing adherence support. CCMDD aims to reduce travel cost, wait time and clinic congestion, and enhance patient adherence and satisfaction [20]. To understand CCMDD PUP use among PLWH, we evaluated factors associated with uptake of a community‐ versus clinic‐based PUP at CCMDD enrolment. We hypothesized that community‐based PUP uptake would be associated with individual factors and perceived CCMDD benefits and challenges.

## 2 METHODS

### 2.1 Setting

This study was conducted in Umlazi, an urban township in KwaZulu‐Natal, South Africa, where ∼88% of the population do not have health insurance and utilize (free) public sector services [21]. Chronic patients attend health facilities monthly, where PLWH on ART wait hours in queues to pick‐up medication with limited clinical assessment time, highlighting potential benefits of CCMDD to differentiate between needs of clinically ill patients and patients established on ART [22].

CCMDD clinical eligibility criteria for PLWH include being non‐pregnant, on ART for ≥1 year and virologically suppressed. Once enrolled, patients select either a community‐ or clinic‐based PUP for collecting their chronic medications every 2–3 months and attend clinic 1–2 times annually for prescription renewal and bloodwork. At clinic‐based PUPs, patients collect from their clinic in a separate, fast‐track lane; at community‐based PUPs (e.g. private pharmacies, schools, churches, home‐based care centres, creches and adherence clubs), patients collect at an external venue that may have a more convenient location or hours than their clinic [23]. At both community‐ and clinic‐based PUPs, the patient presents an appointment card and receives their pre‐packaged medications, without additional clinical support. Patients may attend a facility for clinical assessment at any time, separate from the CCMDD process.

### 2.2 Study design

We analysed cross‐sectional baseline data from an ongoing, prospective observational cohort study evaluating factors associated with patient uptake and clinical outcomes on CCMDD. PLWH ≥18 years who met clinic CCMDD eligibility criteria were enrolled from nine public sector clinics in Umlazi from October 2018 to March 2020, prior to their enrolment in CCMDD. We excluded two clinics that did not offer both PUP types to CCMDD enrolees. At study enrolment, participants provided informed consent and were administered a baseline questionnaire to ascertain demographics and other factors that may influence PUP uptake. Participant clinical data are being collected via records review for 12 months post‐baseline to monitor medication pick‐up, clinic attendance, and virologic and immunologic outcomes. Study data were captured and managed using REDCap [24].

The study was approved by the University of KwaZulu‐Natal Biomedical Research Ethics Committee and Mass General Brigham Institutional Review Board (Protocol 2017‐P‐001690).

### 2.3 Data collection

Participants provided demographic details and answered questions about anticipated CCMDD benefits and challenges of their chosen PUP. We assessed mental health using the five‐item Mental Health Inventory test and calculated mental health composite scores [25]. In addition, we used a 13‐item social support scale to calculate a Social Support Index [26]. We assessed self‐perceived barriers to healthcare using a 12‐question instrument modified from the ARTAS‐II trial and asked eight questions on competing needs in the 6 months prior to enrolment [27, 28]. We assessed perceived self‐efficacy using the 10‐item General Self‐Efficacy Scale, validated to assess a person's belief about their abilities [29, 30]. We assessed internalized HIV stigma using the six‐item Internalized AIDS‐Related Stigma Scale [31] and disclosure concerns (i.e. anticipated stigma if one's serostatus is disclosed) using six questions from the HIV Stigma Scale [32]. We evaluated HIV‐related discrimination (i.e. enacted stigma) with four questions from the People Living with HIV Stigma Index, which asks about frequency of discrimination (being excluded, gossiped about, verbally or physically assaulted) [33].

### 2.4 Statistical analysis

The primary outcome was uptake of community‐based pick‐up through CCMDD. Due to little variability in the number of self‐perceived barriers, competing needs, HIV stigma, disclosure concerns and discrimination, we classified these as “any” or “none.” We considered the four most perceived CCMDD benefits individually and grouped the rest as “other.” The most common perceived challenge was also analysed individually alongside the presence of “any” versus “no” challenges.

We report participant characteristics with descriptive statistics. We used chi‐square and Wilcoxon rank‐sum tests to compare participants electing to use community‐ versus clinic‐based pick‐up. We used univariate and multivariable mixed logistic regression models with clinic as a random factor to assess factors associated with community‐based PUP uptake. Factors with *p*<0.05 in univariate models were included in multivariable models; age and gender were included *a priori*. We describe associations with community‐based PUP uptake using odds ratios, 95% confidence intervals and two‐tailed *p*‐values, with *p*<0.05 indicating statistical significance. One “other” gender response was treated as missing because of the small numbers in this category. Statistical analyses were performed with SAS version 9.4 (SAS Institute, Cary, NC).

## 3 RESULTS AND DISCUSSION

We included 1521 participants from seven clinics. Median age was 36 years (IQR: 30–44), 67% were females, 57% were unemployed and 44% had HIV diagnosed between 2012 and 2016. Additional participant characteristics by PUP are shown in Table 1. Eight hundred and thirty‐one (55%) and 690 (45%) participants elected to use community‐ and clinic‐based pick‐up, respectively. Participants electing to use community‐based pick‐up were slightly younger (35 vs. 37 years, *p*=0.002), were more likely to be employed (*p*=0.002), lived farther from clinic (*p*<0.001), paid more to get to clinic (*p*=0.035), were less likely to walk to clinic (67% vs. 73%, *p*=0.011) or report barriers to care (24% vs. 31%, *p*=0.002), had higher self‐efficacy score (*p*<0.001) and reported more HIV stigma (50% vs. 38%, *p*<0.001) or disclosure concerns (43% vs. 36%, *p*=0.004).

**Table 1:** Participant characteristics (*N* = 1521)

Characteristics	Overall	Community pick‐up point (*n* = 831)	Clinic pick‐up point (*n* = 690)	*p*‐Value
Age, years (median [IQR])	36.0 [30.0–44.0]	35.0 [30.0–43.0]	37.0 [31.0–45.0]	0.002
18–25, *n* (%)	138 (9.1)	83 (10.0)	55 (8.0)	0.039
26–40, *n* (%)	848 (55.8)	478 (57.5)	370 (53.6)	
>40, *n* (%)	535 (35.2)	270 (32.5)	265 (38.4)	
Female, *n* (%)	1012/1520 (66.6)	564/830 (68.0)	448 (64.9)	0.213
Employment status				
Unemployed, *n* (%)	858/1520 (56.5)	435 (52.4)	423/689 (61.4)	0.002
Employed <40 hours per week, *n* (%)	74/1520 (4.9)	42 (5.1)	32/689 (4.6)	
Employed ≥40 hours per week, *n* (%)	588/1520 (38.7)	354 (42.6)	234/689 (34.0)	
Year of HIV diagnosis				
2011 or before, *n* (%)	336/1444 (23.3)	174/805 (21.6)	162/639 (25.4)	0.198
2012–2016, *n* (%)	640/1444 (44.3)	359/805 (44.6)	281/639 (44.0)	
2017 or after, *n* (%)	468/1444 (32.4)	272/805 (33.8)	196/639 (30.7)	
Distance to clinic, km				
<5, *n* (%)	847/1520 (55.7)	412/830 (49.6)	435 (63.0)	<0.001
5–10, *n* (%)	455/1520 (29.9)	262/830 (31.6)	193 (28.0)	
>10, *n* (%)	218/1520 (14.3)	156/830 (18.8)	62 (9.0)	
Time to clinic, minutes				
0–15, *n* (%)	578/1429 (40.5)	311/792 (39.3)	267/637 (41.9)	0.164
16–29, *n* (%)	297/1429 (20.8)	179/792 (22.6)	118/637 (18.5)	
>29, *n* (%)	554/1429 (38.8)	302/792 (38.1)	252/637 (39.6)	
Cost to clinic, Rand				
None, *n* (%)	1084 (71.3)	575 (69.2)	509 (73.8)	0.035
20 or less, *n* (%)	316 (20.8)	193 (23.2)	123 (17.8)	
>20, *n* (%)	121 (8.0)	63 (7.6)	58 (8.4)	
Walked to clinic	1061 (69.8)	557 (67.0)	504 (73.0)	0.011
Mental health score				
<53, *n* (%)	64/1515 (4.2)	36/829 (4.3)	28/686 (4.1)	0.984
53–80, *n* (%)	551/1515 (36.4)	299/829 (36.1)	252/686 (36.7)	
81–96, *n* (%)	633/1515 (41.8)	346/829 (41.7)	287/686 (41.8)	
>96, *n* (%)	267/1515 (17.6)	148/829 (17.9)	119/686 (17.4)	
Low social support, *n* (%)	487/1520 (32.0)	261 (31.4)	226/689 (32.8)	0.562
Any barriers to care, *n* (%)	413 (27.2)	199 (24.0)	214 (31.0)	0.002
Any competing needs, *n* (%)	132 (8.7)	75 (9.0)	57 (8.3)	0.598
Self‐efficacy score				
10–35, *n* (%)	335/1519 (22.1)	159/829 (19.2)	176 (25.5)	<0.001
36–39, *n* (%)	436/1519 (28.7)	212/829 (25.6)	224 (32.5)	
40, *n* (%)	748/1519 (49.2)	458/829 (55.3)	290 (42.0)	
Any HIV stigma, *n* (%)	658/1490 (44.2)	405/818 (49.5)	253/672 (37.7)	<0.001
Any HIV disclosure concerns, *n* (%)	592/1491 (39.7)	351/817 (43.0)	241/674 (35.8)	0.005
Any discrimination, *n* (%)	116 (7.6)	56 (6.7)	60 (8.7)	0.152
Any other chronic medications through CCMDD^a^, *n* (%)	78 (5.1)	41 (4.9)	37 (5.4)	0.706

^a^ Among participants taking chronic medications: 45 had hypertension, 11 had diabetes and 2 had asthma.

The most common perceived CCMDD benefits and challenges are shown in Table 2. Participants electing to use community‐based pick‐up more commonly perceived PUP location (59% vs. 44%, *p*<0.001) or hours (39% vs. 10%, *p*<0.001) as benefits and less commonly perceived shorter PUP queues (68% vs. 80%, *p*<0.001) or visiting clinic less (69% vs. 79%, *p*<0.001) as benefits. These participants also more often indicated other benefits of CCMDD (30% vs. 26%, *p*=0.044), anticipated any challenges (9% vs. 5%, *p*=0.004) and perceived lack of follow‐up for missed medication collection as a challenge (6% vs. 2%, *p*<0.001).

**Table 2:** Common perceived benefits and challenges of CCMDD (*N* = 1521)

	Overall	Community pick‐up point (*n* = 831)	Clinic pick‐up point (*n* = 690)	*p*‐Value
Benefits				
Pick up point in a convenient location	798 (52.5)	492 (59.2)	306 (44.4)	<0.001
Pick up point hours are more convenient	392 (25.8)	325 (39.1)	67 (9.7)	<0.001
Queue would be quicker at pick‐up point	1117 (73.4)	568 (68.4)	549 (79.6)	<0.001
Have to visit clinic less often	1119 (73.6)	575 (69.2)	544 (78.8)	<0.001
Other^a^	429 (28.2)	252 (30.3)	177 (25.7)	0.044
Challenges				
Any perceived challenge^b^	104 (6.8)	71 (8.5)	33 (4.8)	0.004
No proper follow‐up if I miss my collection date	62 (4.1)	47 (5.7)	15 (2.2)	<0.001

^a^ Other benefits included: “It is more expensive to travel to clinic,” “Lower risk for catching diseases from other people at pick‐up points,” “Health workers in clinic are not friendly,” “Anonymity at pick‐up point,” “Can send someone else to pick up medication,” “Other.”

^b^ Perceived challenges included: “No proper follow‐up if I miss my collection date,” “Distance to pick‐up point too far,” “Clinic location more convenient,” “Clinic hours are more convenient,” “Worried people I know might see me at pick‐up point,” “Want to see a healthcare provider to be checked regularly,” “Want to access other services at clinic,” “Need to accompany someone else to clinic,” “Don't trust my medication will be kept safely, or that it is correct one at pick‐up point,” “Other.”

Table 3 shows results of the univariate and multivariable analyses. In multivariable analysis, community‐based PUP uptake was associated with younger age (aOR 1.18 per 10‐year decrease, 95% CI 1.05–1.33), being employed ≥40 hours per week (aOR 1.42, 95% CI 1.10–1.83) versus being unemployed, no self‐perceived barriers to care (aOR 1.42, 95% CI 1.09–1.86) and scoring 36–39 (aOR 1.44, 95% CI 1.03–2.01) or 40 (aOR 1.91, 95% CI 1.39–2.63) versus 10–35 on the self‐efficacy scale. Additional factors included more convenient PUP location (aOR 2.32, 95% CI 1.77–3.04) and hours (aOR 5.09, 95% CI 3.71–6.98) as perceived CCMMD benefits, and lack of in‐clinic follow‐up after missed collection as a perceived challenge (aOR 4.37, 95% CI 2.30–8.31).

**Table 3:** Factors associated with uptake of a community‐based pick‐up point

Characteristic	OR, univariate analyses	*p*‐Value	aOR, multivariable analyses	*p*‐Value
Age^a^	**1.13 [1.02–1.26]**	**0.022**	**1.18 [1.05–1.33]**	**0.005**
Female	1.07 [0.86–1.34]	0.548	1.26 [0.97–1.63]	0.083
Employment status (reference, unemployed)				
Employed <40 hours per week	1.44 [0.88–2.36]	0.148	1.37 [0.81–2.33]	0.239
Employed ≥40 hours per week	**1.48 [1.19–1.84]**	**<0.001**	**1.42 [1.10–1.83]**	**0.007**
No self‐perceived barriers to care	**1.35 [1.06–1.71]**	**0.014**	**1.42 [1.09–1.86]**	**0.010**
Self‐efficacy score (reference, 10–35)				
36–39	**1.41 [1.04–1.91]**	**0.027**	**1.44 [1.03–2.01]**	**0.033**
40	**1.89 [1.43–2.51]**	**<0.001**	**1.91 [1.39–2.63]**	**<0.001**
No discrimination	**1.51 [1.02–2.24]**	**0.040**	1.43 [0.93–2.22]	0.106
Perceived benefits of CCMDD				
Convenient location	**2.42 [1.91–3.06]**	**<0.001**	**2.32 [1.77–3.04]**	**<0.001**
Convenient hours	**5.39 [4.00–7.26]**	**<0.001**	**5.09 [3.71–6.98]**	**<0.001**
Shorter queues	**0.53 [0.42–0.68]**	**<0.001**	0.77 [0.59–1.02]	0.066
Visit clinic less	**0.61 [0.47–0.78]**	**<0.001**	0.79 [0.60–1.04]	0.098
Perceived challenges of CCMDD				
No follow up	**4.07 [2.21–7.50]**	**<0.001**	**4.37 [2.30–8.31]**	**<0.001**

^a^ Per 10‐year decrease.

Bolded values represent *p*‐values <0.05.

In this study, we leveraged the widespread implementation of CCMDD in Umlazi to assess uptake of community‐based pick‐up among patients eligible for CCMDD. Previous studies on DSD have included randomized trials or retrospective analysis [9, 10, 11, 12, 13, 34, 35, 36]. Prospective enrolment allowed a unique opportunity to study factors influencing patient‐level uptake of community‐based ART delivery.

Patients electing for clinic‐based medication pick‐up may face barriers to care as well as low self‐efficacy. While CCMDD aims to reduce service delivery barriers (with shorter clinic waiting times) and structural concerns (with expanded pick‐up location and hour options), persisting barriers undermine the utility of the strategy [4, 5, 6]. Conversely, patient self‐efficacy has traditionally been a facilitator to care; here, efforts to increase self‐efficacy may have downstream effects improving clinic congestion as patients accept community‐based care [6, 37]. To maximize CCMDD's impact, strategies must further address systemic and structural barriers.

Younger age is associated with loss to follow‐up from clinic care and higher attrition from DSD programs, highlighting the need for CCMDD to ensure continued engagement [6, 10, 38, 39]. For older adults, while there is limited knowledge on interventions to improve ART access, strategies must address barriers of stigma, transportation, waiting times and non‐communicable disease service integration commonly experienced by this population [40, 41].

Perceptions of CCMDD focused on convenience, with those identifying location or hours as benefits more likely to choose community‐based pick‐up. Patients valuing convenience and using PUPs accordingly indicates the promise of CCMDD to reduce travel and improve retention in care [6, 42]. Full‐time employment being associated with community‐based PUP uptake is consistent with patient interviews identifying that CCMDD reduced disruption to employment [43]. Increasing number and types of PUPs may further reduce barriers.

Studies on patient preferences between community‐ and clinic‐based ART delivery have found clinic‐based services to be favoured, especially in urban settings [44, 45, 46, 47]. In Johannesburg adherence clubs, patients were more likely to recommend joining if they were in clinic‐ versus community‐based clubs [48]. Similar to our findings, concerns about adequate medical follow‐up influenced these preferences and have been barriers to implementation of Community Drug Distribution Points in Uganda [16, 45, 48, 49]. Concerns about inadequate follow‐up indicate a need to better integrate CCMDD participants with clinic‐based care to avoid loss to follow‐up.

While stigma was not a significant factor in this study, others have suggested that DSD can reduce stigma by allowing patients to avoid being seen in clinic [50, 51]. However, fears of HIV disclosure have also discouraged community medication distribution [45, 48, 49, 50]. Specifically, PLWH were less likely to accept home ART delivery than patients with other chronic conditions due to such fears [52]. Because CCMDD includes medications for a variety of chronic conditions, the risk of HIV stigma may be mitigated; however, confidentiality should be prioritized so patients feel comfortable using community‐based PUPs.

DSD options have been important in minimizing exposures and ensuring continuity of HIV care during Covid‐19 [53, 54]. Mitigation efforts have led to increased eligibility, duration of ART refills and consultations, and community‐based delivery options across sub‐Saharan Africa [55, 56]. Further, providers in Uganda have reported increased patient uptake of community‐based ART compared to before Covid‐19 [56]. Additional research should focus on Covid‐19 effects on patient outcomes in CCMDD.

Our study has several limitations. First, since all participants were enrolled in CCMDD, factors affecting general patient uptake of DSD cannot be determined. Second, certain PUPs were not always available as options to participants. Third, our results represent CCMDD perceptions at enrolment; these may change once patients have experienced medication pick‐up. Finally, the results are from a single urban township, and may not be generalizable to other settings in South Africa, particularly rural settings, where CCMDD use may differ.

## 4 CONCLUSIONS

This multi‐site study assessed the uptake of community‐ versus clinic‐based pick‐up through CCMDD in Umlazi, South Africa. To increase the uptake of community‐based pick‐up, and thus increase patient convenience and decongest overburdened public sector clinics, strategies should be tailored to patients’ age, employment, self‐perceived barriers to care and self‐efficacy. Strategies should also ensure that patients accepting community‐based ART continue to receive the clinical care that they need.

## COMPETING INTERESTS

The authors declare that they have no competing interests.

## AUTHORS’ CONTRIBUTIONS

IVB, SG, ND, ST, LMB and RAP designed the research study. AK, NN, ZS and MN performed the research. BAB, NJW, AS and DZ managed study data and compliance. JY and RAP analysed the data. IVB and AS wrote the paper. All authors have read and approved the final manuscript.

## FUNDING

This work was funded by the National Institute of Mental Health (R01 MH114997, IVB), the Weissman Family Massachusetts General Hospital Research Scholar Award (IVB) and philanthropic funding from the Graham Family.

## DISCLAIMER

These contents are solely the responsibility of the authors and do not necessarily represent the official views of the National Institutes of Health or the Massachusetts General Hospital Executive Committee on Research.

## Data Availability

Data are available upon reasonable request to the corresponding author.
